# Housing price gradient and immigrant population: Data from the Italian real estate market

**DOI:** 10.1016/j.dib.2017.12.018

**Published:** 2017-12-15

**Authors:** Valentina Antoniucci, Giuliano Marella

**Affiliations:** Department of Civil, Environmental and Architectural Engineering, University of Padova, via Venezia 1, 35131 Padua, Italy

**Keywords:** Housing market, Immigrants, Multivariate regression, Real estate market, Price gradient

## Abstract

The database presented here was collected by Antoniucci and Marella to analyze the correlation between the housing price gradient and the immigrant population in Italy during 2016. It may also be useful in other statistical analyses, be they on the real estate market or in another branches of social science. The data sample relates to 112 Italian provincial capitals. It provides accurate information on urban structure, and specifically on urban density. The two most significant variables are original indicators constructed from official data sources: the housing price gradient, or the ratio between average prices in the center and suburbs by city; and building density, which is the average number of housing units per residential building. The housing price gradient is calculated for the two residential sub-markets, new-build and existing units, providing an original and detailed sample of the Italian residential market. Rather than average prices, the housing price gradient helps to identify potential divergences in residential market trends.

As well as house prices, two other data clusters are considered: socio-economic variables, which provide a framework of each city, in terms of demographic and economic information; and various data on urban structure, which are rarely included in the same database.

**Specifications**

**Table:** 

Subject area	*Economics*
More specific subject area	*Real estate*
Type of data	*Table*
How data was acquired	*Survey*
Data format	*Raw*
Experimental factors	*Sample pretreatment as follows: sources with incomplete data were rejected. The variables surveyed were examined using ordinal scales. Data were transformed for three variables on a logarithmic scale.*
Experimental features	*First descriptive statistics were provided, and a correlation matrix was prepared. Then a multivariate regression was performed in three stages, testing the correlations between center-periphery price gradients and immigrant populations, as well as other socio-economic features.*
Data source location	*The data were collected from 112 Italian provincial capitals, distributed all over the country.*
Data accessibility	*The data are attached to this article.*

**Value of the data**

• The database provides an original indicator of center-periphery housing price gradients (based on official sources), for both existing and new-build units, in the most important Italian cities. To our knowledge, this is the largest dataset available on these features of house prices in Italy.
• Another original indicator - building density - was constructed (again based on official data sources) to describe the urban structure of Italian cities.
• The data presented here can be processed by means of a variety of statistical methods, from multivariate regression to cluster analysis, and hedonic price models.
• In addition to the housing price gradient, the raw data provide socio-economic information on Italy's major cities that could be used for research in the whole field of social science, not only in real estate analyses.

## 1 Data

The data relate to 112 Italian provincial capitals (i.e. almost all of them) all over the country. The data are divided into three thematic clusters, as shown in Table 1: housing price gradients for new-build and existing units; data representing urban-level socio-economic features; and data on urban morphology and structure.

**Table 1:** Variables.

**Cluster**	**Variables**	**Measure scale**	**Coding system**
*Prices*	Housing gradient (new units)	Ratio	No.
	Housing gradient (existing units)		
*Socio-economic features*	Population	Interval	No.
	Immigrants	Interval	No.
	Employees in retail and tourism	Interval	No.
	Employees in services	Interval	No.
	Female employment rate	Interval	Percentage
	Employment rate	Interval	Percentage
	Per capita income	Interval	Euro
*Urban structure features*	Urban density	Ratio	Pop/km^2^
	Per capita public transport availability rate	Ratio	No.
	Distance between center and suburbs	Interval	km
	Housing unit surface area	Interval	m^2^
	Mean altitude	Interval	m a.s.l.
	Total housing units	Interval	No.
	Building density (housing units per residential building)	Ratio	No.

It is worth mentioning that housing price data, and especially transaction prices, are inadequately collected in Italian research and practice. Although the Italian Inland Revenue Agency records housing transactions, these data are not publicly available. Our housing price gradient was consequently constructed from prices quoted in other official sources. All the data on prices and population characteristics relate to the year 2015, while the data on urban structure refer to 2011, when the latest National Census on buildings was conducted. The data from 2011 still provide an accurate description of the country's residential building heritage [1] due to the stagnation of the building sector in Italy in the last nine years (the number of residential construction permits issued dropped by 80.8% from 2005 to 2013 [2], [3], [4], [5], [6], [7]). The measurement scale adopted (ordinal and ratio) is consistent with the literature [see for instance [8]]. The coding systems adopted for the variables were dictated by those available from the sources.

## 2 Experimental design, materials and methods

The 112 cities chosen for the survey account for all but one of Italy's provincial capitals. One small town in Sardinia was not included in the dataset. The sample was chosen to provide a nationwide overview of the correlation between housing price gradient [9] and immigrant population [10] resident in the cities. The housing price gradient was calculated as the ratio between the average housing prices in the city centers and the suburbs. This clearly provides a simplified representation of urban structure (especially for larger cities), but most Italian cities are still consistent with the monocentric urban model. The distinction between two sub-markets (new-build and existing units) was maintained because of the difference in their long-term trends, which is a characteristic of the Italian housing market [11], [12]. Considering the housing price gradient, instead of average prices, may help to reveal a divergence in housing prices, which can represent social polarization phenomena *within* a city, not just *between* cities [13]. All socio-economic variables were obtained from the Italian Statistics Institute (ISTAT), except for per capita income, which was provided by the Bank of Italy. Demographic and economic characteristics of urban populations are crucial to any analysis on real estate, due to the marked variability of factors affecting housing price trends. The per capita public transport availability rates were provided by an Italian research center on regional development. The number of resident immigrants and the immigrant proportion of a city's population are useful, also for the purpose of analyzing ethnic and urban segregation [14]. Four variables were used to control for the economic vitality of cities: two, employment rate and female employment rate, have been widely used in the literature [15]; the other two, employees in retail and tourism, and in services, help to identify the prevalent economic sector – given the high incidence of tourism in many Italian cities with an important historical heritage [16], [17], [18]. Lastly, per capita income is traditionally one of the variables positively correlating with both average house prices and housing price gradients. This variable, like the population and number of immigrants, was indicated on a logarithmic scale. It is particularly useful in regression analyses on real estate [19], [20], [21], [22].

The last cluster of variables considers, among other urban structure features, the distance between center and suburbs (which represents the administrative size of a given city), and the mean altitude (as a city's morphology could significantly affect house price trends). The two main measures of urban density considered were: population density, expressed as the number of inhabitants per square kilometer; and building density, or the average number of housing units per residential building. The latter is the most accurate measure of urban density in real estate analyses, especially in countries like Italy where urban developments vary significantly across the country. The surface area of housing units and the total number of housing units in a city, combined with the previous variables, refines the description of urban density at diverse stages, while the per capita transportation availability rate is needed to see whether a city has a sprawling or compact layout (the use of public transport is more common in denser and bigger cities).
